# COVID-19 Vaccine Survey among Healthcare Workers. A Community Experience.

**DOI:** 10.51894/001c.35628

**Published:** 2022-09-06

**Authors:** Nikita Theophilus, Carlos Rios-Bedoya, Ghassan Bachuwa

**Affiliations:** 1 Internal Medicine Hurley Medical Center

**Keywords:** COVID-19, Covid-19 vaccination, Vaccine Hesitancy, Covid-19 vaccine Survey

## Abstract

**INTRODUCTION:**

In December 2019, the coronavirus (COVID-19) caused by severe acute respiratory syndrome coronavirus 2 (SARS-CoV-2) made its first appearance in Wuhan, China with a pandemic declared by March 2020. As the death toll continued to rise, the Centers for Diseases Control and Prevention (CDC) recommended healthcare workers to strongly encourage the general population to receive COVID-19 vaccinations. For this to be effective, it is important to understand the general perceptions of the health care workers and persons associated with the healthcare industry towards their acceptance of the vaccine.

**METHODS:**

The authors of this 2021 cross-sectional study administered a 28-item survey to a convenience sample of 1,257 (43.1%) healthcare system workers out of a total of 2,915. The survey assessed respondents’ demographic information, COVID-19 vaccine status, work-related exposures to COVID-19, reasons for receiving or refusing the vaccine, and primary sources of vaccine related information. Respondents were classified as vaccine status/intention positive or negative.

**RESULTS:**

Those in the youngest 18 - 35 years age group were significantly less likely to receive the vaccine (p < 0.01) and male healthcare workers were significantly more likely to receive the vaccine (p = 0.01). White respondents, 759 (77.9%) were also more likely to receive the vaccine than African-American, 127 (13%). It was more likely for persons to be vaccinated when encouraged/provided (p = 0.01) information by their respective employers. A subgroup of 277 (22.0%) respondents reported their employer as the primary source of vaccine information, causing the authors to conclude that employer information was the most influential informational factor impacting COVID-19 vaccination.

**CONCLUSION:**

Vaccine hesitancy continues to be a major obstacle hampering the success of COVID-19 vaccination promotion programs. Results indicate that a combination of a prior COVID-19 diagnosis, information dispensed by a person’s employer, persons’ home living situations, and contact with persons who had an uneventful post vaccination experience increased the likelihood of vaccination.

## INTRODUCTION

The coronavirus 2019 (COVID-19) pandemic is an ongoing pandemic caused by severe acute respiratory syndrome coronavirus 2 (SARS-CoV-2). The virus first appeared in Wuhan, China in December 2019 and was declared to be a pandemic by March 2020. By June 2021, the world had recorded over 176 million cases, with over 3.8 million deaths primarily attributable to COVID-19.^1^

As mortality and morbidity rates have increased worldwide, researchers have worked to better understand the pathology of the virus. Unfortunately, evolving preventive and treatment measures have not reached a definitive conclusion, resulting in global social and economic disruptions.^2–5^ In addition to the development of effective hospital treatment regimens, proven vaccines have now been formulated to prevent severe COVID-19 illness from occurring.^6,7^

As COVID-19 pandemic rates have increased, as of publication of this article, there have been over 461 million cumulative cases and over 6 million worldwide deaths.^6^ Although various clinical trials have shown vaccines to be highly effective, skepticism and negative public opinions and hesitancy towards COVID-19 vaccination continues to be an ongoing hurdle.^7–9^ Vaccine Hesitancy appears to be one of the major overall obstacles hampering the success of most vaccination programs.^7–10^ Studies have also demonstrated only a 60-67% acceptance of COVID-19 vaccination among US healthcare workers due to lack of trust in the vaccine, insufficient information, and other personal beliefs.^10,11^

In 2020, the CDC officially encouraged all healthcare workers and the general population to receive an available COVID-19 vaccination.^12^ However, examining the varied perceptions of the healthcare workers towards COVID-19 vaccines remains a vital component of public health vaccination programs.^13^

### Study Objective

The objective of this 2021 cross-sectional survey study was to investigate the primary factors influencing COVID-19 vaccine acceptance and hesitancy in a convenience sample of mid-Michigan healthcare workers.

## METHODS

### Study Design

The authors utilized a quantitative cross-sectional study design. After IRB approval in February 2021, a survey invitation via email was sent to all healthcare worker employees at a hospital medical center in Flint, Michigan. Healthcare workers were defined as all healthcare personnel employed by the healthcare system who were directly or indirectly involved in patient care. A total of 1,258 (43.1%) responses were collected from a solicited 2,915 documented healthcare workers over a period of 21 days.

The survey that was developed by the authors consisted of 28 multiple choice and open-ended questions (Appendix I). Survey items asked respondents to report their socio-demographic characteristics (e.g., Age, Gender affiliation, Racial/Ethnicity Affiliation, etc.) and personal clinical information (i.e., possible pregnancy, medical comorbidities, COVID-19 vaccine status, work-related exposure to COVID-19, reasons for getting or not the vaccine, knowledge about the vaccine, and sources of information influencing vaccination decisions). Quantitative survey item data results were analyzed by second author CRB.

### Selected Study Outcome

The vaccination status of each healthcare worker respondent was determined from their responses to a series of multiple-choice questions regarding their current vaccination status or intention to get vaccinated. The question “Will you take the vaccine?” was paired with the following response options for those answering “Yes”: “I have already taken the vaccine”, “No”, and “Not sure”. Those answering the first two options (i.e., “Yes” and “I have already taken the vaccine” at least three months ago) were classified as “vaccine status/intention positive” while those answering the other two options were classified as “vaccine status/intention negative.”

### Statistical Analyses

Before conducting any inferential statistical analysis, the authors assessed data distribution outliers (i.e., out of range values), and completed data cleaning to review frequencies, proportions, descriptive statistics and figures (e.g., histograms and box and whisker plots).

Bivariate correlation analytic procedures were conducted to determine any associations between the study explanatory variables (i.e., (categorized) Age Group, Gender Affiliation, Racial/Ethnicity Affiliation, COVID-19 exposure level, primary source of COVID-19 information, intention to get vaccinated, etc.) and self-reported COVID-19 vaccination status/intention. Such analyses included Chi Square and Fisher exact tests.

Multivariate logistic regression analytic procedures were also conducted to examine any relationship between study explanatory variables (i.e., Age Group, Gender Affiliation, COVID-19 exposure level, primary source of COVID-19 information, intention to get vaccinated, etc.) and the main selected study outcome (i.e., COVID-19 vaccination status/intention). All analyses were conducted by author CRB using the Stata statistical software package (Stata Corporation, College Station, TX). The usual 0.05 Type I error threshold for statistical significance was observed when interpreting results.

## RESULTS

A total of 1,257 (43.1%) healthcare workers replied to the survey out of 2,915 questionnaire invitations. Of those who replied, 965 (76.8%) respondents had already received their first COVID-19 vaccine dose. As shown in Table 1, bivariate analyses showed that categorized Age Group was significantly associated with vaccine status (p < 0.01). Male healthcare workers were also significantly more likely to receive the vaccine than females (p = 0.01). White respondents were also more likely to receive the vaccine than African -Americans (p = 0.01).

**Table 1.** Predictors of Sample Healthcare Workers’ COVID-19 Vaccination Status/Intention.

	Total Sample n=1,248 (%)	Non-Vaccinated n=247 (%)	Vaccinated n=1,001 (%)	p-value
Age Group (%) 18-35 36-50 51-65 >65	294 (23.6) 479 (38.4) 428 (34.3) 47 (3.8)	69 (27.9) 114 (46.2) 60 (24.3) 4 (1.6)	225 (22.5) 365 (36.5) 368 (36.8) 43 (4.3)	**< 0.01**
Gender Affiliation (%) Male	282 (22.7)	40 (16.6)	242 (24.2)	**0.01**
Race/Ethnicity Affiliation (%) White African American Other More than one group	927 (76.6) 178 (14.7) 64 (5.3) 41 (3.4)	168 (71.2) 52 (21.6) 9 (3.8) 8 (3.4)	759 (77.9) 127 (13.0) 55 (5.7) 33 (3.4)	**0.01**
Education Completed (%) High School or less Some College Bachelor’s Degree Graduate Degree	34 (2.8) 451 (36.5) 474 (38.4) 276 (22.4)	7 (2.9) 110 (44.9) 98 (40.0) 30 (12.2)	27 (2.7) 341 (34.4) 376 (38.0) 246 (24.9)	**< 0.01**
Prior COVID-19 Diagnosis (%) Yes	136 (10.9)	39 (15.9)	97 (9.7)	**0.01**
COVID-19 Exposure Frequency (%) Every day/Every other day Weekly/Twice a week Every other week Uncertain	255 (20.8) 124 (10.1) 53 (4.3) 793 (64.7)	39 (16.3) 25 (10.5) 7 (2.9) 168 (70.3)	216 (21.9) 99 (10.0) 46 (4.7) 625 (63.4)	0.13
Perceived Level of COVID-19 Concern (%) No concern Low Medium High	157 (12.7) 491 (39.6) 351 (28.3) 242 (19.5)	69 (28.1) 94 (38.2) 52 (21.1) 31 (12.6)	88 (8.8) 397 (39.9) 299 (30.1) 211 (21.2)	**< 0.01**
Household Composition (%) Significant other & children Parents and children Children only Living alone Living with 2+ people	723 (58.3) 40 (3.2) 170 (13.7) 152 (12.3) 155 (12.5)	147 (59.8) 3 (1.2) 45 (18.3) 18 (7.3) 33 (13.4)	576 (58.0) 37 (3.7) 125 (12.6) 134 (13.5) 122 (12.3)	**< 0.01**
Number of Chronic Health Conditions (mean ±SD)	0.34 (±0.79)	0.31 (±0.79)	0.34 (±0.79)	0.60
Primary Source of Vaccine Information (%) Academic/Scientific Articles Health Care Workers Employer Family/Friends Mainstream/Social Media Other	353 (28.5) 294 (23.8) 272 (22.0) 15 (1.2) 255 (20.6) 48 (3.9)	71 (29.1) 53 (21.7) 39 (16.0) 1 (0.4) 56 (23.0) 24 (9.8)	282 (28.4) 241 (24.3) 233 (23.5) 14 (1.4) 199 (20.0) 24 (2.4)	**< 0.01**

Statistically significant p values appear in **bolded font**. Percentages may not add up to 100 because of rounding.

Table 1 demonstrates several other factors (e.g., prior COVID-19 diagnosis, living with children, etc.) that significantly influenced each respondent’s vaccination status. However, possessing multiple medical comorbidities (e.g., Hypertension, Diabetes, Thyroid disorders, Cardiac or Pulmonary comorbidities, etc.) was not a significant factor influencing vaccination status.

Table 2 shows results of our adjusted multivariate logistic regression estimates for factors hypothesized to be associated with COVID-19 vaccination status. For example, Males were 1.8 times more likely (adj. OR = 1.8; 95% CI: 1.1, 2.7; p = 0.01) to be vaccinated after controlling for the other variables in the table. Similarly, African Americans were 60% less likely (p < 0.01) to have been vaccinated at time of survey than Whites. The survey was administered over a period of three weeks in February 2021.

**Table 2.** Characteristics Independently Influencing COVID-19 Vaccination Status

**Factor**	**Odds Ratio (95% CI)**	**p-value**
**Age Group** 18-35 36-50 ** 51-65** >65	Reference 1.3 (0.9, 1.9) 2.4 (1.6, 3.8) 2.0 (0.6, 6.2)	0.20 **< 0.01** 0.23
**Gender Affiliation** (Male)	1.8 (1.1, 2.7)	**0.01**
**Race/Ethnicity Affiliation** White **African-American** Other More than one race/ethnic group	Reference 0.4 (0.3, 0.7) 1.2 (0.5, 2.7) 0.7 (0.3, 1.7)	**< 0.01** 0.74 0.39
**Completed Education** Graduate Degree **Bachelor’s Degree ** ** Some College** High School or less	Reference 0.4 (0.3, 0.7) 0.3 (0.2, 0.6) 0.3 (0.1, 1.0)	**0.01** **< 0.01** 0.05
**Household composition** Significant Other and children **Parents/Parents and children** Children only Living alone Living with 2 or more people	Reference 4.8 (1.4, 16.8) 0.8 (0.5, 1.3) 1.9 (1.1, 3.5) 1.1 (0.7, 1.8)	**0.01** 0.46 0.03 0.77
**Prior COVID-19 Diagnosis?** Yes	0.5 (0.3, 0.8)	**< 0.01**
**Perceived Level of COVID-19 Concern** No concern Low Medium High	Reference 2.9 (1.8, 4.5) 4.1 (2.5, 6.6) 6.2 (3.5, 11.1)	**< 0.01** **< 0.01** **< 0.01**
**Primary Source of Vaccine Information** Academic/Scientific Articles Health Care Workers Employer Family/Friends Mainstream/Social Media ** Other**	Reference 1.1 (0.7, 1.7) 1.5 (0.9, 2.4) 3.3 (0.4, 28.0) 0.9 (0.6, 1.5) 0.3 (0.1, 0.6)	0.67 0.13 0.27 0.77 **< 0.01**

*multivariate logistic regression analysis.

Statistically significant factors and p values appear in **bolded font**.

Healthcare workers with less than a graduate degree were significantly less likely to have received the COVID-19 vaccine at the time of the survey. Other factors significantly related with vaccination status were a previous COVID-19 diagnosis (p < 0.01) and perceived level of concern about COVID-19. Compared to no concern, an upward trend was observed by level of concern; low level (p < 0.01), medium level (p < 0.01), and high level (p < 0.01). (Table 2)

## DISCUSSION

Due to their increased relative COVID-19 risks, healthcare workers were generally the first recipients of the COVID-19 vaccination program.^14,15^ Similar to our study results, Race/ethnicity affiliation has been found to be a significant factor influencing the phenomenon of vaccine hesitancy, with 83.0% of African-Americans across the U.S. being less receptive to receiving the vaccine.^11^

We found from our survey results that the reported frequency and risk of exposure to the COVID-19 virus had no impact on healthcare workers’ likelihood of getting vaccinated. However, respondents’ household composition sometimes served as a statistically significant influencing role. (Table 2) Our survey results are consistent with findings noted in other studies where respondents were more likely to be vaccinated if they were in contact with a person who had received the vaccination and had not suffered significant side effects.^16^

As shown in Table 1, 272 (22.0%) of respondents indicated their employer as their primary source of vaccine information. Unfortunately, we were unable to precisely define “Other” sources from the way the survey had been developed. Based on the available data from the survey, the conclusion is that employers in the community may prove to be a key source of vaccine guidance to either promote or discourage vaccination. In addition, the Supreme Court passing a limited mandate on health care workers requiring vaccination as of January 2022 may help accelerate vaccination in persons who originally planned to delay or were contemplating vaccination due to increased economic risks from unemployment.^17^

Vaccine Hesitancy appears to be one of the major obstacles hampering the success of most vaccination programs.^7–11^ Vaccine hesitancy is considered to be a delay in acceptance or refusal of vaccination despite availability of vaccination services. Vaccination acceptance is considered a complex decision-making process that involves three factors, which include complacence, confidence, and convenience.^18^ As per the article published, confidence is defined as trust in (i) the effectiveness and safety of vaccines; (ii) the system that delivers them, including the reliability and competence of the health services and health professionals and (iii) the motivations of policymakers who decide on the needed vaccines.^18^

A 2007 meta-analysis conducted regarding a person’s behavior towards the concept of vaccination shows that people perceive the risk of vaccination in two dimensions.^19^ The first one being the perceived likelihood of harm if no action is taken compared to the perceived consequences of side effects from the vaccination itself.^19.^

In more recent studies it has been demonstrated that decision making, and perceived risks are often influenced by health care professionals, government, and/or public health institutions.^20,21^ Individual’s decisions and hesitancy towards various vaccinations have been intricately linked to social, emotional, political, and cultural beliefs.^22^ Our results suggest that the strategic provision of information from workers’ employers and health officials may serve to increase the likelihood of healthcare workers and others to get vaccinated.

### Study limitations

Our three-week cross-sectional survey study was conducted with a local convenience sample of healthcare workers to examine current factors influencing vaccine hesitancy levels. Worker opinions may have changed as more people received the vaccine without incident. Our survey could not identify all specific reasons influencing respondents’ vaccine opinions as most simply choose an option of “Other” response without offering further detailed comments.

## CONCLUSION

These results indicate that vaccine hesitancy remains one of the major obstacles to our nation’s implementation of COVID-19 vaccination programs. The information obtained from a person’s employer, their personal characteristics, and contact with persons who have had an uneventful post COVID-19 vaccination course may in many cases serve to increase healthcare workers’ likelihood of getting vaccinated.

### Conflict of Interest

None

## APPENDIX I

1. What age group do you fall under?
  - 18- 35 years
  - 36- 50 years
  - 51- 65 years
  - > 65 years
2. Which ethnicity do you belong to?
  - White
  - African American
  - Asian
  - Hispanic
  - Native American
  - Other
  - Prefer not to answer
3. What is your relationship status?
  - Single
  - Married
  - Widowed
  - Living with someone
  - Prefer not to answer
4. Do you have roommates or family at home? Mark all that apply
  - I live alone
  - Children
  - Parents
  - Significant other
  - Other family members
  - Room mates
5. What is your highest level of Education?
  - Less than high school
  - Some high school
  - Some college
  - Bachelor’s degree
  - Graduate degree
6. What is your sex?
  - Female
  - Male
  - Prefer not to say
7. Are you currently Pregnant or lactating?
  - Yes
  - No
8. Do you have any chronic medical Conditions?
  - Yes
  - No
9. Check all that apply:
  - Hypertension
  - Diabetes Mellitus
  - Asthma
  - COPD
  - Heart related disease
  - Thyroid disorders
  - Clotting disorders
  - Cancer
  - Obesity
  - On immunosuppressive therapy
  - Other
  - Do not wish to specify
10. How often do you fall ill? (This includes any minor conditions like the common cold, allergy flare ups, etc. that may or may not require a visit to a doctor’s office)
  - Every month
  - Every other month
  - 2-3 times a year
  - Once every other year or lesser
11. Have you ever been admitted to the hospital in the past or required a visit to the Emergency Department due to medical conditions? (excluding normal pregnancies without complications, minor cuts and wounds due to trauma that required an emergency room visit of less than 3 hours from the time of evaluation):
  - Zero
  - Once
  - 1-3 times
  - > 3 times
12. Which sector of the hospital do you work at?
  - Emergency department
  - ICU
  - Step down Intensive unit
  - Floor services
  - Ambulatory Care
  - Administrative Office
  - Floating (moving between units)
  - Confined to departments of indirect patient care (Eg. IT personnel, Kitchen, Laundry, Electrical)
  - Security
13. How often are you exposed to COVID-19 positive patients?
  - Everyday
  - Every other day
  - Twice a week
  - Weekly
  - Every other week
  - Uncertain
14. Have you ever been diagnosed with COVID-19?
  - Yes
  - No
15. What are your levels of concern about getting a serious case of COVID-19 that may require oxygen support, ventilator support, multi-organ failure, proving to be fatal or near fatal?
  - High
  - Medium
  - Low
  - No concern
16. Have you had a relative or a close friend diagnosed with COVID-19?
  - Yes
  - No
17. Did you receive the Flu shot last year?
  - Yes
  - No
18. What is your primary source of vaccine related information?
  - Mainstream media, Radio, Television
  - Social media (Facebook, Twitter, etc.)
  - Family, friends
  - Health care workers
  - Residents, Physicians
  - Employer
  - Academic/scientific articles
  - Other
19. When would you be most comfortable with taking the vaccine?
  - Sometime this month
  - After 1 month
  - After 2 months
  - After 3 months
  - Uncertain
  - I’ve already taken the vaccine
20. Do you know anyone who has been vaccinated?
  - Yes
  - No
21. Will you take the vaccine?
  - Yes
  - No not sure
  - I’ve already taken the vaccine
22. What is your primary reason for taking the vaccine?
  - It is recommended
  - Because it decreases the chance of illness
  - Because of pandemic fatigue
  - To protect my loved ones due to my higher risk of exposure
23. Would you take the vaccine even after hearing about the new virus strain associated with COVID-19?
  - Yes
  - No
  - Maybe
24. Did you always plan on taking the vaccine?
  - Yes
  - No
  - Uncertain
25. What changed your mind about taking the vaccine?
  - Coworkers, friends, family were taking the vaccine
  - A figure head of yours was taking the vaccine
  - Peer Pressure
  - Other
26. What is your primary reason to refuse vaccination?
  - Worried about short-term side effects
  - Worried about long-term side effects
  - Anxiety about the new vaccine
  - Short duration of vaccine process compared to standard
  - Believe the vaccination process is politically driven
  - Uncertain benefits
  - Diagnosed to be COVID-19 positive
  - Not studied with Pregnancy
  - Not studied with Lactation
  - Not studied with Immunocompromised states
  - Religious beliefs
  - Personal medical history
  - Other
27. Would you recommend the vaccine to family/friends who work in a health care setting?
  - Yes
  - No
28. Would you recommend the vaccine to family/friends who don’t work in a healthcare setting?
  - Yes
  - No
